# Ultraviolet GaN Light-Emitting Diodes with Porous-AlGaN Reflectors

**DOI:** 10.1038/s41598-017-05391-0

**Published:** 2017-07-10

**Authors:** Feng-Hsu Fan, Zun-Yao Syu, Chia-Jung Wu, Zhong-Jie Yang, Bo-Song Huang, Guan-Jhong Wang, Yung-Sen Lin, Hsiang Chen, Chyuan Hauer Kao, Chia-Feng Lin

**Affiliations:** 1 Department of Materials Science and Engineering, National Chung Hsing University, Taichung, 402 Taiwan; 2 Department of Photonics, Feng Chia University, 100, Wenhwa Road, Seatwen, Taichung 40724 Taiwan; 3 Department of Applied Materials and Optoelectronic Engineering, National Chi Nan University, Nantou County, Taiwan; 4 Department of Electronics Engineering, Chang Gung University, Kwei-Shan, Tao Yuan Taiwan

## Abstract

A GaN/AlGaN ultraviolet light emitting diode (UV-LED) structure with a porous AlGaN reflector structure has been demonstrated. Inside the UV-LED, the n^+^-AlGaN/undoped-AlGaN stack structure was transformed into a porous-AlGaN/undoped-AlGaN stack structure through a doping-selective electrochemical etching process. The reflectivity of the porous AlGaN reflector was 93% at 374 nm with a stop-bandwidth of 35 nm. In an angle-dependent reflectance measurement, the central wavelength of the porous AlGaN reflector had blueshift phenomenon by increasing light-incident angle from 10° to 50°. A cut-off wavelength was observed at 349 nm due to the material absorption of the porous-AlGaN/u-AlGaN stack structure. In the treated UV-LED structure, the photoluminescence emission wavelength was measured at 362 nm with a 106° divergent angle covered by the porous-AlGaN reflector. The light output power of the treated UV-LED structure was higher than that of the non-treated UV-LED structure due to the high light reflectance on the embedded porous AlGaN reflector.

## Introduction

Gallium nitride (GaN) materials have been applied in optoelectronic devices such as light-emitting diodes (LEDs), laser diodes (LDs)^1^, and vertical cavity surface emitting lasers (VCSELs)^2, 3^. Ultraviolet LEDs (UV-LEDs) at 365 nm emission wavelength with potential to replace the conventional Hg lamp are currently used for curing, document verification, and plant growth. In addition, the epitaxial AlGaN/GaN stacks^4, 5^ and AlN/GaN stacks^6, 7^ structures have been reported for the bottom distributed Bragg reflectors (DBRs) in GaN-based VCSEL devices. Large lattice mismatch and low refractive index difference in the stack structures are the challenges for the epitaxial DBR structures with long epitaxial growth time. To realize the high reflectivity with less pairs of stack structure, the air-gap/GaN DBR structures with large refractive index different have been fabricated through selectively anodized processes^8–10^ and thermal decomposition techniques^11–13^. However, the low mechanical strength and the tiny high reflective area of the air-gap/GaN DBR structure remain challenges for the photonic device fabrication. Plawsky *et al*.^14^ reported the nanoporous material for the photonics through the evaporation-induced self-assembly process and oblique or glancing angle deposition. GaN epitaxial layers were grown on the Si substrate with the embedded Y_2_O_3_/Si^15^, Gd_2_O_3_/Si^16^, AlN/GaN^17^, and AlN/AlGaN^18^ DBR structures. Berger *et al*.^19^ reported the narrow-band distributed Bragg reflectors realized by GaN:Ge modulation-doped structure. The embedded distributed Bragg reflector^20, 21^, the high reflective tin-doped indium oxide/Ag nano-dots/Al-based reflectors^22^, the Ti_3_O_5_/Al_2_O_3_ DBR^23^, and the ITO/dielectric DBR^24^ were demonstrated to enhance the light extraction process in the UV-LED structures. Furthermore, nanoporous GaN materials with low effective refractive index have been proposed for the DBR structure applications^25–27^.

In this paper, a GaN/Al_0.04_GaN UV-LED structure with a porous Al_0.085_GaN reflector was fabricated through the selective electrochemical (EC) etching process. The Si-doped AlGaN/undoped-AlGaN stack structure inside the device was transformed into the porous-AlGaN/undoped-AlGaN stack structure functioning as an embedded reflector. The EC-treated porous AlGaN reflector with an 8.5% Al content didn’t absorb the electroluminescence (EL) emission light from the GaN/AlGaN active layer. The EL emission light at 361 nm from the GaN/AlGaN active layer could be reflected by the bottom porous-AlGaN reflector exempted from the light absorption of the bottom unintentionally doped GaN layer and GaN buffer layers. High light reflectance of the porous-AlGaN reflector was formed at the bottom of the GaN/AlGaN active layer so that the light extraction efficiency could be improved. Moreover, optical properties of the UV-LED structure with and without porous-AlGaN reflector were analyzed in detail.

## Results

The LED epitaxial layer consisted of a 30-nm-thick GaN buffer layer grown at 530 °C, a 2.0-µm-thick unintentionally doped GaN layer (u-GaN, 1050 °C, 5 × 10^16^ cm^−3^), twelve pairs of n^+^-Al_0.085_GaN:Si/u-Al_0.085_GaN stack structure (n^+^-AlGaN, 1050 °C, 1 × 10^19^ cm^−3^), a 30 nm-thick undoped-Al_0.04_GaN layer (1050 °C), a 3.0-µm-thick n-Al_0.04_GaN layer (1050 °C, 2 × 10^18^ cm^−3^), ten pairs of GaN/Al_0.04_GaN (3 nm/12 nm) multiple-quantum wells (MQWs, 900 °C), a 30 nm-thick p-type Al_0.04_GaN:Mg layer (1050 °C, 1 × 10^18^ cm^−3^), and a 10 nm-thick p-type GaN:Mg layer (1050 °C, 2 × 10^18^ cm^−3^). The SiH_4_ source was used as a n-type doping source during the epitaxial growth of the n^+^-AlGaN layers with an high electron concentration about 1 × 10^19^ cm^−3^ so that the n^+^-AlGaN layers were transformed into the porous AlGaN layers in the porous reflector structure. The parallel wet etching channels on the UV-LED wafer were formed through a laser scribing (LS) process to reach the as-grown 12-period n^+^-AlGaN/u-AlGaN stack structure by using a 355 nm pulse laser. The Si-heavily doped n^+^-AlGaN:Si layer was transformed into a porous AlGaN layer through the doping-selective electrochemical etching process in a 0.5 M nitride acid solution at a positive external bias voltage of 12 V^28^. After the EC-etching process, a high refractive index n-type AlGaN:Si layer was transformed into a low refractive index porous AlGaN layer. Then, a 200-nm-thick indium tin oxide (ITO) film was deposited on the mesa region functioning as a transparent conductive layer. The ITO layers on the p-type GaN:Mg layer were annealed in furnace at 600 °C for 20 min to improve the ohmic contact property. The dimension of the UV-LED device was 50 μm × 50 μm in size with ITO conductive layer. Then, the patterned Ti/Al (50 nm/200 nm) metal layers were deposited on the bottom n-type GaN:Si conductive layer for the n-type contact metal pad.

**Figure 1:**
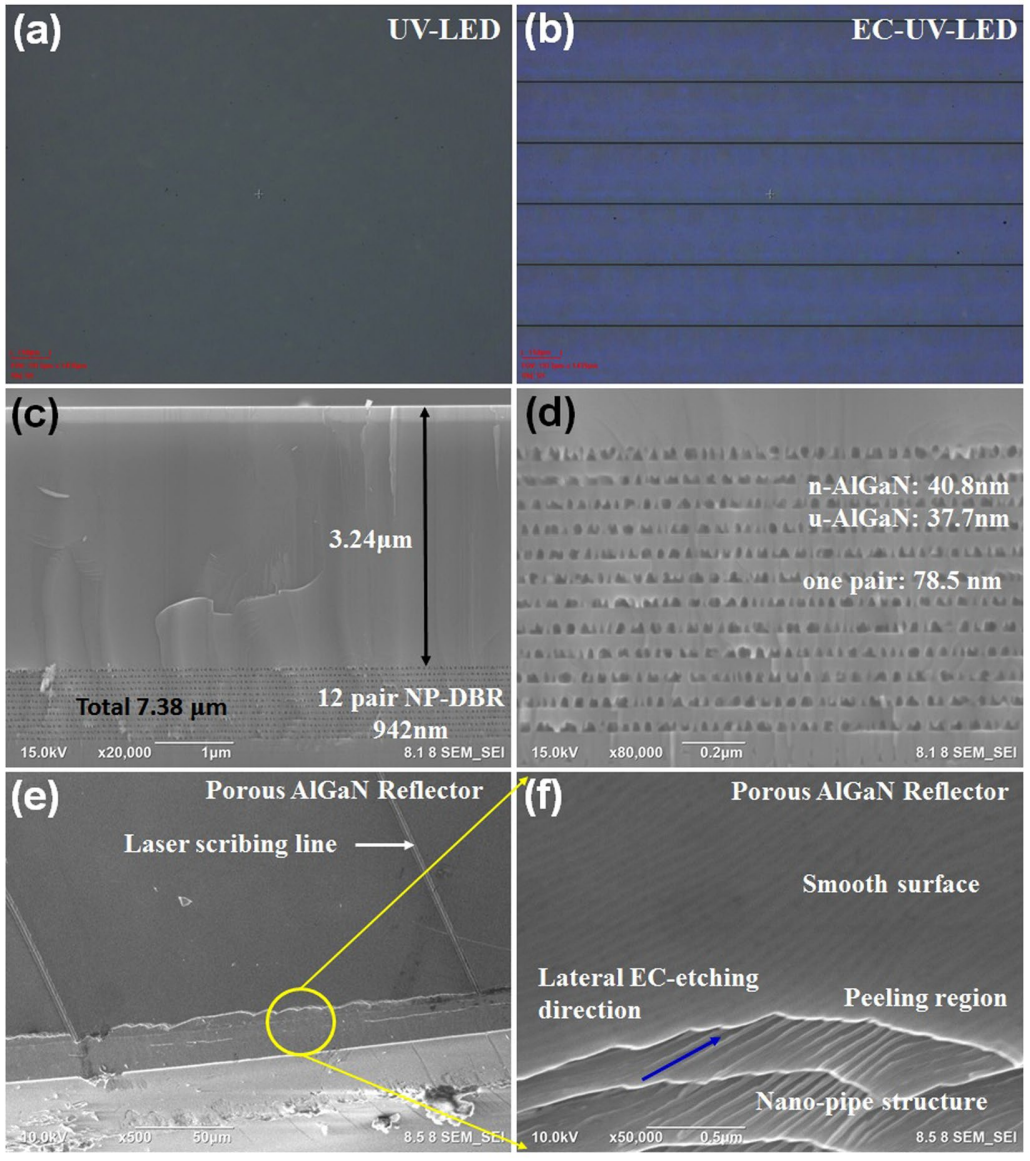
OM images of the (**a**) UV-LED and (**b**) EC-UV-LED were observed. (**c**) The cross-sectional SEM micrograph of the EC-UV-LED structure was observed. (**d**) A 40.8nm-thick porous-AlGaN layer and 37.7nm-thick u-AlGaN layer were measured in the 12-pair reflector structure. SEM images of the porous AlGaN reflector structure were observed at (**e**) the cleaved region and (**f**) the peeling region.

The OM images of the non-treated UV-LED and the EC-UV-LED were observed in Fig. 1(a) and (b). The flat and smooth surface was observed in the non-treated UV-LED as shown in Fig. 1(a). The n^+^-AlGaN/u-AlGaN stack structure embedded in the UV-LED structure was exposed in the etching solution through the laser scribing channels. After the EC etching process, a colorful image was observed on the surface of the EC-UV-LED structure as shown in Fig. 1(b) due to the light reflection from the porous-AlGaN reflector under the OM light. The electrochemical etching channels were defined through the laser scribing (LS) process. The parallel laser scribed lines were observed in Fig. 1(b). The electrochemical etching process were occurred from two sides of the LS lines and merged at the central LS defined regions. The etching fronts were perpendicular to the LS lines that defined as the lateral etching process. In Fig. 1(c), the top 3.24 μm-thick UV-LED structure and the bottom porous-AlGaN reflector were observed in the cross-sectional SEM micrograph. Twelve-pair porous-AlGaN/u-AlGaN stack structure consisted of a 40.8 nm-thick porous-AlGaN layer and a 37.7 nm-thick u-AlGaN layer as shown in Fig. 1(d). The n^+^-AlGaN:Si epitaxial layers were etched as the porous AlGaN layers in the porous-AlGaN/u-AlGaN stack structure. Moreover, the porous-AlGaN/undoped-AlGaN periodic structure was observed clearly due to the high Si-doping-selectively EC etching process on the n^+^-AlGaN:Si layers. The plan-view SEM images of the porous AlGaN reflector without the top LED structure is shown in Fig. 1(e). The laser scribing lines, the cleaved region, and the peeling-off region of the porous AlGaN reflector were observed as shown in Fig. 1(e). In Fig. 1(f), the smooth top surface of the u-AlGaN layer were observed without the EC-etching process. The porous AlGaN structures were viewed in the porous AlGaN reflector. The layer-by-layer structure was slightly separated due to the sample preparation for the SEM observation. After the EC-etching process, the pipe structures were observed at the n^+^-AlGaN layers and the direction of the pipe structure was along with the EC-etching direction. The embedded pipe structure was observed clearly through the top u-AlGaN layer with a smooth surface.

**Figure 2:**
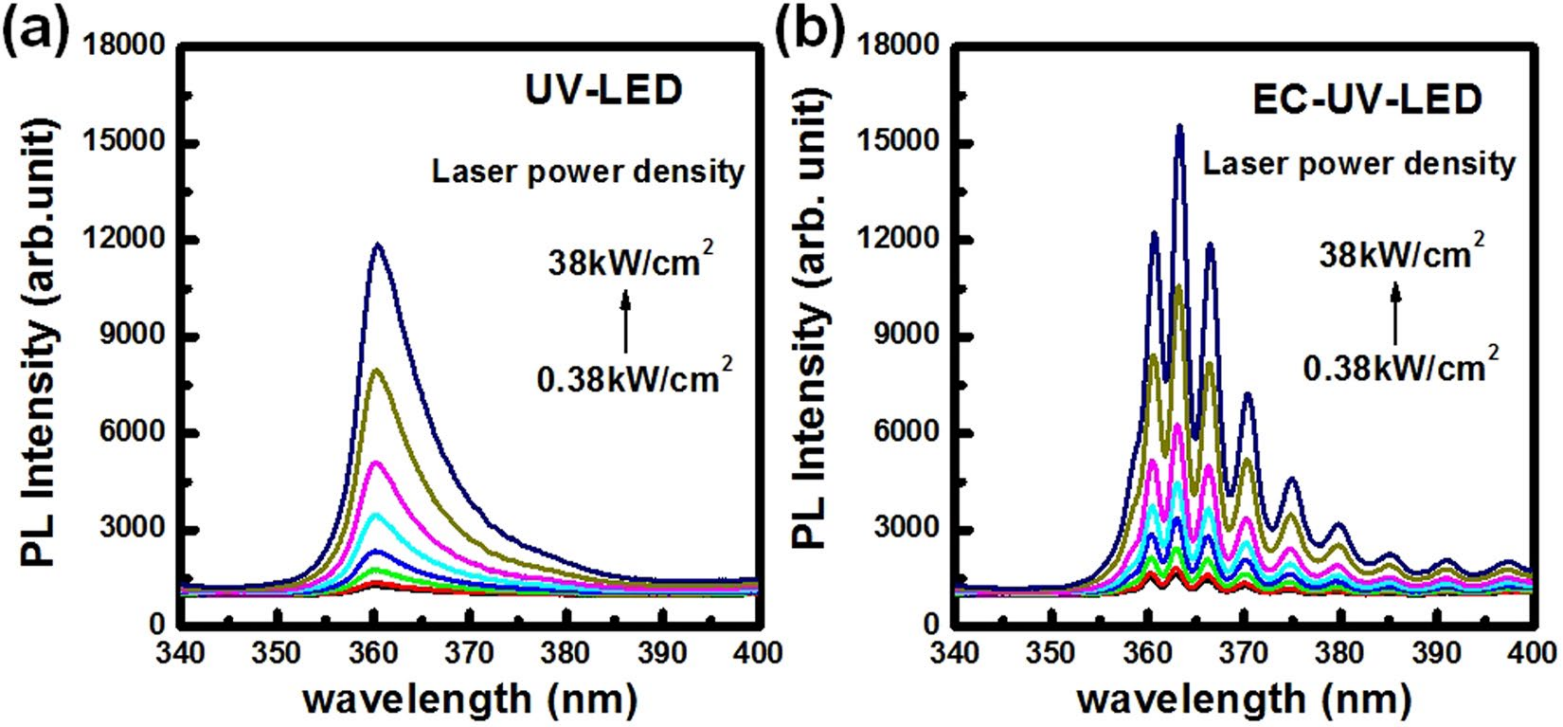
Power-dependent μ-PL spectra of the (**a**) UV-LED and (**b**) EC-UV-LED were measured at room temperature by varying the laser excited power density of the 325 nm HeCd laser.

In Fig. 2, the micro-PL spectra of non-treated and treated LED structures were measured by using the 325 nm HeCd laser as the excited laser source through a 15× objective lens with a 10 μm-diameter laser spot. The laser power densities were varied from 0.38 kW/cm^2^ to 38 kW/cm^2^ for the power dependent PL measurement. The PL peak wavelengths were measured at 361 nm for the UV-LED structure and at 364 nm for the EC-UV-LED structure, respectively. The slight interference phenomenon of the PL spectrum was observed in the UV-LED structure between the top Air/GaN:Mg and the bottom GaN/Al_2_O_3_ flat interfaces. Strong light interference of the PL spectrum was observed in the EC-UV-LED structure compared with the non-treated UV-LED because of the cavity effect of the UV-LED structure above the porous-AlGaN reflector. This strong light interference of the EC-UV-LED structure implied the high reflectivity on the embedded porous-AlGaN reflector.

**Figure 3:**
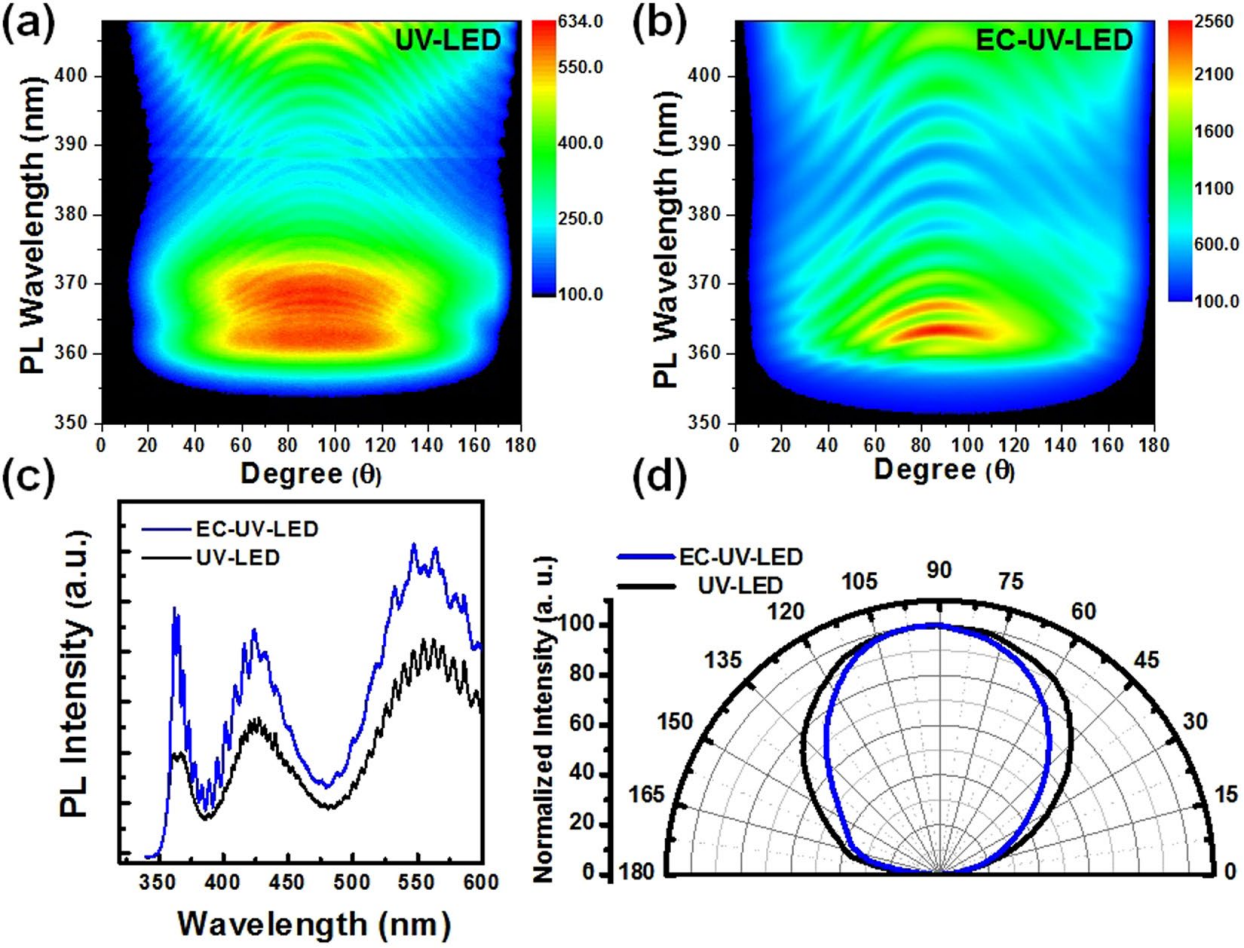
PL emission spectra of (**a**) the UV-LED and (**b**) the EC-UV-LED were measured through the angle-resolved PL measurements using a 325 nm diode laser as an excitation laser source. (**c**) The PL spectra of both of the LED structures were measured at normal direction (at 90°). (**d**) Normalized PL far-field radiation pattern of both of the LED structures were measured.

In Fig. 3(a), the angle-dependent PL spectra were measured from the regular PL setup with the 325 nm HeCd laser illuminated on the sample with a 45° incident angle and a 200 μm-diameter laser spot size (low laser excited power density). The laser spot size was reduced from 1 mm to 0.2 mm by using a diaphragm. In the UV-LED, the fringes in the angle-dependent PL spectra were observed clearly due to the light interference between the top air/AlGaN and the bottom AlGaN/sapphire interfaces. The PL spectra was measured at the front-side of the flat LED wafer without chip process. In Fig. 3(a), the Fabry–Pérot (FP) interference line-patterns were observed in the UV-LED caused by the light interference at the top air/GaN:Mg and bottom GaN/sapphire interface. In Fig. 3(b), the PL far-field radiation pattern with low density interference phenomenon was observed in the EC-UV-LED structure. The broad band emission spectra with the interference phenomenon were observed in both of the LED structures. Moreover, The PL peak wavelength of the non-treated UV-LED was measured at 362.3 nm for the GaN active layer, 369.2 nm for the 3.0-µm-thick n-type AlGaN:Si layer, 428 nm for the GaN:Mg layer, and 562 nm for the yellow band emission peak, respectively, as shown in Fig. 3(c). By formation of the porous-AlGaN reflector below the GaN/AlGaN active layer, the PL emission intensity of the EC-UV-LED was enhanced compared with the non-treated UV-LED structure. Based on the interference phenomenon, the PL far-field radiation pattern could be used to calculate the detailed dimensions inside the UV-LED structure. Therefore, the thickness of the epitaxial layer could be computed as the value of 6.18 μm from the interference pattern. In the EC-UV-LED structure, the thickness of the LED structure between the top air/GaN interface and the bottom porous-AlGaN reflector was about 3.24 μm, which was calculated from PL far-field radiation pattern. The reason of the reduction of the light optical path in the EC-UV-LED structure was the light confinement between the porous-AlGaN reflector and air. Normalized PL far-field radiation patterns of both of the LED structures were observed in Fig. 3(d). By formation of the embedded porous-AlGaN reflector, the divergent angle of the EC-UV-LED (at 106°) was slightly reduced compared with the non-treated UV-LED structure (at 126°). The PL emission light from the GaN/AlGaN MQW active layer could be reflected by the embedded porous-AlGaN reflector so that the emission divergent angle could be slightly reduced.

**Figure 4:**
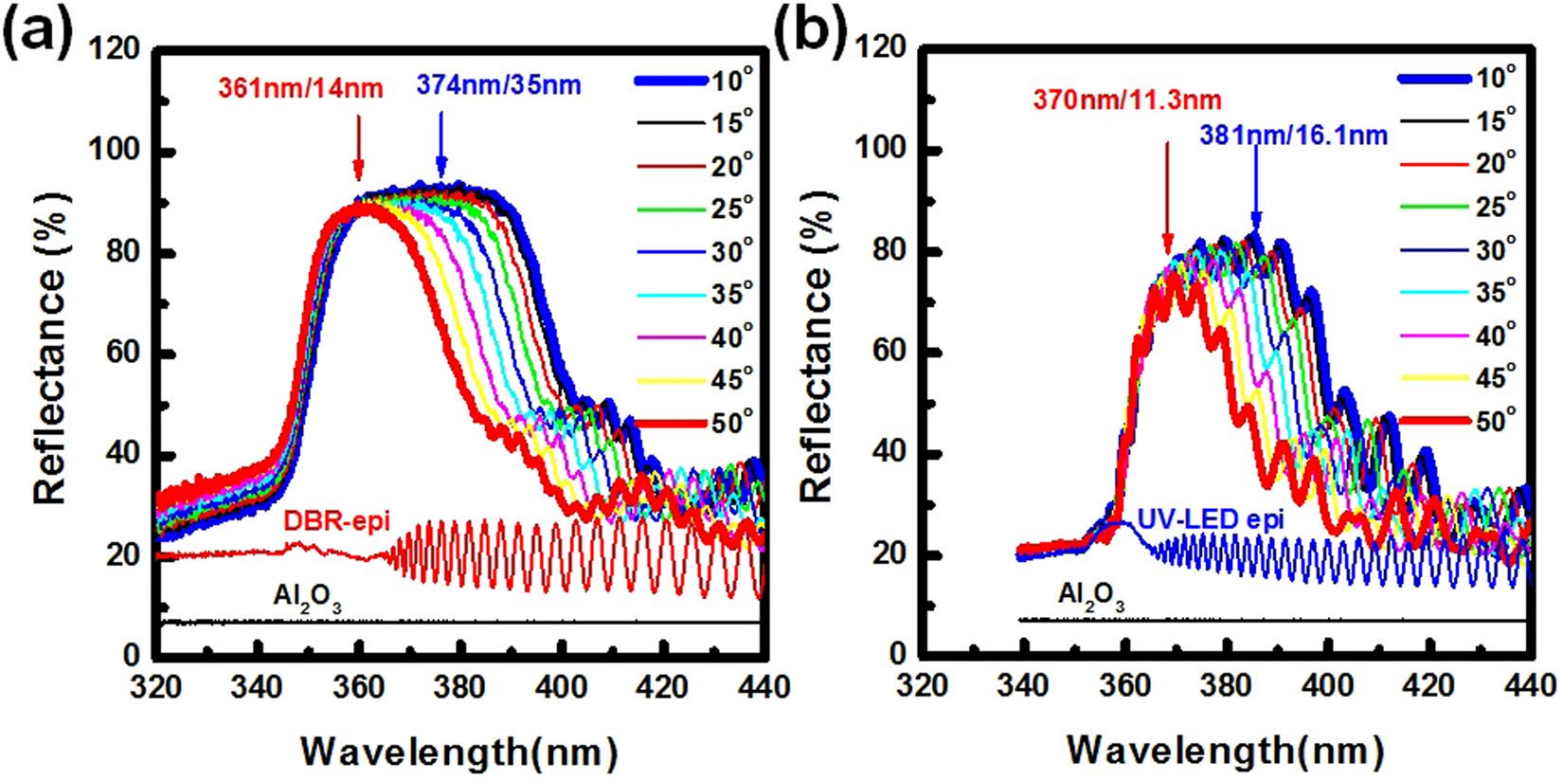
Angle-dependent reflectance spectra of (**a**) the porous AlGaN reflector and (**b**) the EC-UV-LED structures were measured by varying the detected angles from 10° to 50°. The central wavelength and the band-width at 10° and 50° detected angles were labeled. The reflectance spectra of the non-treated DBR epitaxial structure (DBR-epi), the non-treated UV-LED epitaxial structure (UV-LED epi), and the flat Al_2_O_3_ substrate were measured.

The angle-dependent reflectance spectra of the UV reflector and the EC-UV LED with UV reflector were measured by varying the detected angles from 10° to 50°. In Fig. 4(a), the UV reflector was measured as the values of 374 nm for central wavelength and 35 nm for the band-width at 10° detected angle. When the detected angle increased to 50°, the UV reflector was measured at 361 nm for central wavelength and 14 nm for the band-width. The reflectance spectra of the non-treated DBR epitaxial structure, the non-treated UV-LED epitaxial structure, and the flat Al_2_O_3_ substrate were measured in Fig. 4, respectively. The reflectivity of the flat Al_2_O_3_ substrate was about 7.2%. This value was close to the theoretical value of 7.9% which the refractive index of the Al_2_O_3_ material was about 1.78. By increasing the light incident angle, the optical path difference (OPD) of the reflected light was reduced in the porous-AlGaN/u-AlGaN reflector. The central wavelength of the porous-reflector at large light incident angle (50°) was shifted to a shorter wavelength compared with it at small light incident angle (10°)^29^. The cut-off wavelength of the reflectance spectra was observed at 349 nm due to the material absorption of the n^+^-AlGaN/u-AlGaN stack structure with 8.5% Al content. At a detected angle of 10°, the peak reflectivity of the porous-AlGaN reflector was about 93% at 374 nm with 35 nm-width smooth stopband in the reflectance spectrum.

In the EC-UV LED structure, a 3.24 μm-thick UV-LED epitaxial structure was grown on n^+^-AlGaN/u-AlGaN stack structure. After the EC etching process, the porous-AlGaN/u-AlGaN stack structure was formed below the UV-LED structure functioning as an embedded reflector. The angle-dependent reflectance spectra of the EC-UV LED structure were measured as shown in Fig. 4(b). In the EC-UV-LED structure, the light interference phenomenon was observed in the reflectance spectrum due to the light reflection between the top Air/GaN:Mg interface and the bottom AlGaN/porous-reflector interface. From the angle-dependent reflectance spectra, the cut-off wavelength of the EC-UV-LED was obtained at 360 nm due to the light absorption in the UV-LED structure above the porous-AlGaN reflector. The reflectivity of the porous-AlGaN reflector (93%, 374 nm) was higher than that of the EC-UV-LED structure (83%, 381 nm). The phenomenon was caused by the light absorption and the light reflection on the top UV-LED structure in the EC-UV-LED structure. In Fig. 4(b), high light reflectance was achieved at 361 nm in the EC-UV-LED structure at different incident angles from 10° to 50°. The 361 nm light emitted from the GaN/AlGaN MQW active layers could be reflected by the embedded reflector with the high reflectance which covered wide incident angles. From the far-field radiation measurement, the divergent angle of the EC-UV-LED was about 106° so that most part of the emission light could be reflected by the porous AlGaN-reflector at different incident angles. The PL emission intensity of the EC-UV-LED structure was enhanced compared with that of the non-treated UV-LED structure as shown in Fig. 3(c). After the EC etching process, the n^+^-AlGaN layers in the reflector structure were transformed into the porous-AlGaN layer. The effective refractive index of the bottom AlGaN layer (porous-AlGaN/u-AlGaN stack structure) was reduced and increased the light reflection. In Fig. 3(c), the high PL emission intensity of the EC-UV-LED beyond 400 nm wavelength was observed because the high emission intensity was caused by the light reflection and the light scattering on the bottom treated AlGaN layer.

**Figure 5:**
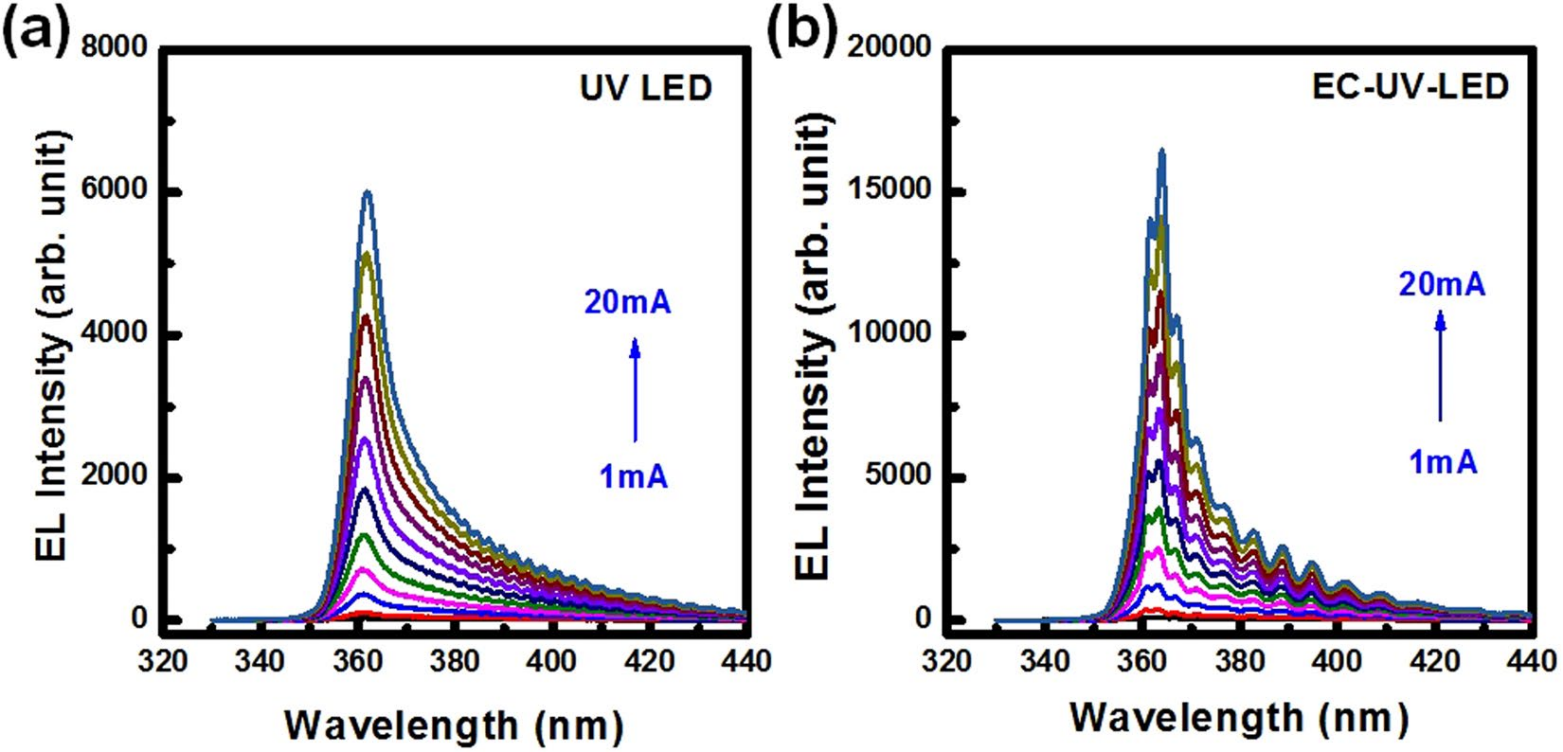
The EL emission spectra of the (**a**) UV-LED and (**b**) EC-UV-LED were measured by varying the injection current at room temperature.

The EL spectra of the two distinct LED structures were measured by varying the injection current from 1 mA to 20 mA, as shown in Fig. 5(a) for the UV-LED and in Fig. 5(b) for the EC-UV-LED, respectively. In Fig. 5(a), the EL emission wavelength of the non-treated UV-LED was observed at about 361.9 nm which had a high density of the FP interference in the EL emission spectrum. The embedded 12-period n^+^-AlGaN/u-AlGaN stack structure was etched as the porous-AlGaN/u-AlGaN stack structure on the sapphire substrate without the sapphire lifted off process. The porous size in the porous-AlGaN reflector was not uniform due to the wet chemical etching process. The reflectivity of the reflector was distributed uniformly on the treated region indicative of the effective refractive index of the porous-AlGaN distributed uniformly in the porous-AlGaN/u-AlGaN stack structure. With the formation of the porous reflector structure, the EL emission wavelengths of the EC-UV-LED were located at around 363.2 nm owing to the high light reflectance on the porous-AlGaN reflector. The central wavelength of porous-AlGaN reflector that enhanced the EL emission intensity at long emission wavelength region was measured at 374 nm with 35 nm line-width at 10° detected angle. The high reflectance spectrum of the porous-AlGaN reflector could cover the EL emission spectrum and enhance the light output power in the EC-UV-LED structure. The FP interference of the EL spectra were also detected in the EC-UV-LED structure due to the light reflection between the top air/GaN:Mg interface and the flat bottom AlGaN/porous-reflector interface.

**Figure 6:**
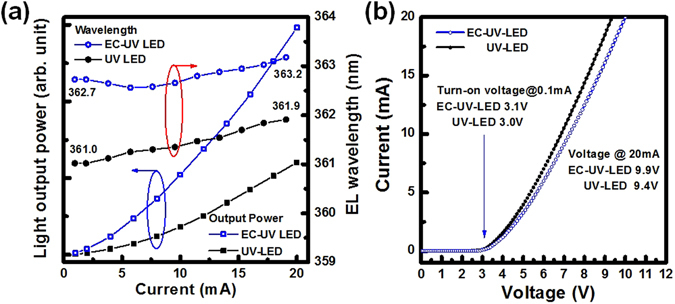
(**a**) Light output power and the peak wavelength of the EL spectra of both of the LED structures were measured. After the embedded reflector structure was formed, the EL emission peak wavelengths of the EC-UV LED were slightly redshifted compared with the non-treated UV-LED structure. (**b**) The I–V curves and the turn-on voltage of both devices are measured.

In Fig. 6(a), the light output power and the EL emission wavelength of both LED structures were measured by varying the injection current. The EL emission intensity of the EC-UV-LED structure was stronger than that of the UV-LED structure. At 20 mA, the peak wavelengths of the EL spectra were measured at 361.9 nm for the UV-LED and 363.2 nm for the EC-UV-LED, respectively. In the EC-UV-LED structure, the peak EL emission wavelength had a slightly redshifted phenomenon caused by formation of the embedded porous-AlGaN reflector structure. Therefore, the light output power of the EC-UV-LED structure was enhanced because of the high light reflectance on the bottom porous reflector structure. In both devices, the current as a function of the operation voltage is shown in Fig. 6(b). The voltages at 0.1 mA (turn-on voltage)/20 mA operating current were measured at 3.0 V/9.4 V for the UV-LED and at 3.1 V/9.9 V for the EC-UV-LED, respectively. The turn-on voltages of both devices were almost the same at an operating current of 0.1 mA. At an operation current of 20 mA, the operation voltage of the EC-UV-LED was slightly higher than that of the UV-LED because the n^+^-AlGaN layers with lower conductivity were transformed into the porous–AlGaN layers with higher resistance in the EC-UV-LED structure.

## Discussion

GaN/AlGaN ultraviolet light emitting diodes with the EC-treated porous-AlGaN reflectors were fabricated. The n^+^-AlGaN/undoped-AlGaN stack structure was transformed into the porous-AlGaN/u-AlGaN structure. Therefore, the reflectivity of the porous AlGaN reflector (93% at 374 nm) was higher than that of the EC-UV-LED (83% at 381 nm) with the top LED active layer. The cut-off wavelengths in the reflectance spectra were obtained at 349 nm for UV reflector and at 360 nm for EC-UV-LED structure related to the light absorption of the AlGaN and the GaN layers. The light output power of the EC-UV-LED structure was higher than that of the UV-LED structure because of the high light reflectance of the embedded porous-AlGaN reflector. The UV-LED structure with the high reflectance porous-AlGaN reflector has potential for the future high efficiency UV optoelectronic device applications.

## Methods

### Epitaxial growth

UV-LED structures were grown on a 2 in. optical-grade c-face (0001) sapphire substrate using a metal organic chemical vapor deposition system. Trimethylgallium (TMGa), trimethylaluminum (TMAl), and ammonia (NH_3_) were used as gallium (Ga), aluminum (Al), and nitrogen (N) sources material, respectively. Silane (SiH_4_) and biscyclopentadienyl magnesium (CP_2_Mg) were used as the n-type doping and p-type doping sources, respectively.

### Electrochemical etch process

An external dc bias was fixed at a positive voltage of 12 V that was applied on the n-type AlGaN:Si layer surface as an anode contact without immersing in a 0.5 M nitride acid solution. A platinum (Pt) electrode was used as the cathode for the electrochemical etch process. The Si-heavily doped n^+^-AlGaN:Si layer was transformed into a porous AlGaN layer through the doping-selective electrochemical etching process. The electrochemical etching channels on the samples were defined through the laser scribing (LS) process. The parallel laser scribed lines were observed in Fig. 1(b). The electrochemical etching process was occurred from two sides of the LS lines and merged at the central mesa regions. The etching fronts were perpendicular to the LS lines defined as the lateral etching process.

### Optical characterization

The surface morphologies of the LED structures were detected by using optical microscopy (OM) and a field-emission scanning electron microscope (FE-SEM, JEOL 6700F). The photoluminescence (PL) spectra of far-field radiation patterns and the electroluminescence (EL) spectra were measured by using monochromator (JOBIN YVON iHR550) with a TE-cooled charge-coupled device (CCD) detector. The light output power and the emission wavelength of both LED structures were measured at normal direction and analyzed through the monochromator.
